# Cancer-specific Regulation of Metabolic and Epigenetic Pathways by Dietary Phytochemicals

**DOI:** 10.1007/s11095-025-03898-0

**Published:** 2025-08-04

**Authors:** Yuxin Pan, Rebecca Mary Peter, Pochung Chou, Parv Dushyant Dave, Jiawei Xu, Ahmad Shanner, Md Shahid Sarwar, Ah-Ng Kong

**Affiliations:** 1 Department of Pharmaceutics, Center for Phytochemical Epigenome Studies, Ernest Mario School of Pharmacy, The State University of New Jersey, Rutgers Piscataway, NJ 08854 USA; 2 Graduate Program of Pharmaceutical Sciences, Ernest Mario School of Pharmacy, The State University of New Jersey, RutgersPiscataway, NJ 08854 USA; 3 Department of Pharmacy, Noakhali Science and Technology University, Sonapur, Noakhali 3814 Bangladesh

**Keywords:** Breast cancer, Cancer prevention, Colorectal cancer, Dietary phytochemicals, Epigenetics, Lung cancer, Metabolism, Prostate cancer, Skin cancer

## Abstract

**Background:**

Cancer is a complex disease triggered by a combination of genetic mutations, metabolic reprogramming, epigenetic modifications, and environmental factors, and is one of the leading health burdens worldwide. Dietary phytochemical are effective in reducing the incidence of cancer by regulating epigenetic and metabolic pathways and possess great potential in cancer prevention and treatment.

**Objectives:**

This review aims to summarize the relevant metabolic and epigenetic changes in cancer, recent research progress in the mechanism study of dietary phytochemicals by regulating key pathways and provides new insights for further research.

**Methods:**

We searched the relevant literature by searching through common databases, such as PubMed, Medline, ScienceDirect, Google Scholar and Web of Science, and summarized the anticancer effects of potential dietary phytochemicals regulating epigenetic and metabolic pathways in common cancers, such as colorectal cancer, prostate cancer, lung cancer, skin cancer, and breast cancer.

**Results:**

Natural phytochemicals not only regulate key pathways, such as DNA methylation, histone modifications, oxidative stress, inflammation, and metabolic reprogramming, but also demonstrate multi-targeted intervention in tumor therapy by inducing apoptosis, inhibiting cell proliferation, angiogenesis, and metastasis. Additionally, the potential of dietary phytochemicals to reverse metabolic and epigenetic changes makes them promising as adjunctive or alternative therapies.

**Conclusion:**

Phytochemicals exhibit significant effects in cancer prevention and treatment by targeting metabolic and epigenetic regulatory networks. Future research should further explore their mechanisms, clinical translational value, and synergistic effects with traditional therapies to provide theoretical foundations and practical guidance for developing new cancer intervention strategies.

## Introduction

Cancer is a complex disease caused by multiple factors, such as genetic mutations, altered cellular metabolism, epigenetic modifications, and the environment [1, 2]. According to Global Cancer Statistics, the proportional number of new cancers worldwide for the year of 2022 was 20 million cases, and the number of deaths due to cancer was as high as 9.7 million [3]. In recent years, lung cancer, the most common as well as the most lethal cancer, has led to nearly 2.5 million new cases, which is one eighth of all cancers worldwide (12.4% of all cancers globally), followed by colorectal, prostate, and gastric cancers [4]. Although research into cancer prevention, diagnosis and treatment continues to increase, demographically based projections suggest that by 2050, the number of new cancer cases will reach 35 million and the cancer burden will increase several folds.

The alteration of cellular mechanisms in carcinogenesis is a process that lasts for several years [5]. When the normal process of cell growth and proliferation is disturbed by genetic mutation or external environment i.e., carcinogenesis initiation, the process is further influenced by various biological signals that ultimately lead to tumor promotion and progression [6]. Conventional surgery and chemotherapy are unable to overcome the problems of irreversible organ damage, toxicity, and drug resistance [2, 7]. Numerous studies have shown that fruits, vegetables and grains consumed by people on a daily basis have been found to be enriched with more than 7,000 dietary phytochemicals, such as glucosinolates and sulforaphane (SFN) in broccoli and kale, epigallocatechin gallate (EGCG) in tea and genistein flavonoids (GFs) in legumes, among others [8–10]. These active phytochemicals have been closely linked to reduced incidence of several cancers. Various *in vivo* and *in vitro* studies have shown that they can effectively modulate various molecular targets and signaling pathways to reverse metabolic and epigenetic alterations during early-stage in cancer cells, including scavenging of oxygen free radicals, modulation of membrane receptors, and enzyme activities such as DNA methyltransferase (DNMTs) [11]. This implies that long-term intake of natural products and their secondary metabolites can intercept cancers prior to the onset of clinical symptoms, inhibit tumor cell survival, proliferation, metastasis, and angiogenesis, i.e., have the potential to reduce cancer incidence [12–16].

Traditional herbal medicines that have been used for thousands of years like Chinese medicine, Ayurvedic medicine (India), and modern phytotherapy in Europe and the United States [6, 17]. Flavonoids, phenols, alkaloids, nitrogen-containing compounds, organosulfur compounds, phytosterols, and carotenoids comprise the mainstay of the cancer-prevention properties [18–25]. According to statistics, the number of approved drugs for use in the treatment of cancer has been increasing [13, 14]. Interestingly, many of the small molecule drugs approved for cancer treatment are based on natural products derived from dietary plants, and research on phytochemicals' role in resisting oxidative stress and inflammatory effects, altering detoxification enzymes and hormone metabolism, and regulating cancer cell proliferation signals has become a new promising avenue in dealing with cancer [11, 26, 27].

Unlike heredity, epigenetic reprogramming refers to reversible alterations in chromatin structure and gene expression status that do not involve changes in the DNA sequence [28]. As a result of the interaction between heredity and environmental factors, epigenetic inheritance can modulate genome-associated functions at multiple levels, including selective transcriptional expression, such as DNA methylation, histone modification and chromosome remodeling as well as post-transcriptional expression regulation of genes, such as noncoding RNAs, transposable elements, among others [10–12, 29]. On the other hand, the phenomenon of altering cellular metabolism in response to various external stimulations is known as metabolic reprogramming, a process prevalent in cancer development and progression [30]. Cellular metabolic enzymes, upstream regulatory molecules, and downstream metabolites change their properties from normal metabolism to aerobic glycolysis (the Warburg effect), altered glutamine metabolism, and altered lipid synthesis, all of which provide the energy and biosynthesis required by rapidly dividing tumor cells providing the required energy and biosynthetic substrates [30–32]. In cancer, metabolic reprogramming and epigenetics interact in a bidirectional manner to drive oncogenesis, enabling tumor cells to avoid normal physiological regulatory functions, resist apoptosis, and continue to proliferate and grow [28]. Metabolites from different metabolic pathways act as substrates, cofactors, agonists, or antagonists of epigenetic modifiers that affect chromatin structure and gene expression [33, 34]. Similarly, abnormal epigenetic regulation alters the expression of key metabolic enzymes and metabolic genes, leading to enhanced metabolic reprogramming that sustains tumor growth. In this review, we will discuss the metabolic and epigenetic changes and their interactions in cancer and will focus on the metabolic reprogramming and epigenetic reprogramming functions of dietary phytochemicals and discuss their potential interceptive roles against major cancer types.

## Overview of Metabolic Pathways and Epigenetic Reprogramming in Cancer

Cancer occurs in organisms in which epigenetic traits are often dysregulated [35]. DNA methylation, the most widely studied epigenetic process, has been described as the transfer of a methyl group to the 5′ position of 5′-CpG-3′ dinucleotide endocytosine (5-mC), catalyzed by the DNA methyltransferases (DNMT1, DNMT3a, and DNMT3b) [35, 36]. In the 1980 s, various studies of changes in DNA methylation patterns began to show that such changes control the binding of transcription factors that affect tumor progression and metastasis [37]. Methylation of CpG islands at the promoter site has been observed in different tumor cells and has become one of the hallmarks of many cancers because of its ability to silence a number of tumor suppressor genes, including P15INK4b, P16INK4a, P14ARF, CDH1, and other genes [38, 39]. In addition, hypomethylation of DNA can activate oncogenes, leading to re-expression of silenced genes, decreased chromosomal stability, and even overall DNA methylation loss in repetitive sequences.

Another important mechanism of epigenetic regulation, histone modification, is often abnormal in cancer as well. In normal cells, histone modifications are made by post-translationally modifying histones (PTMs) to chemically alter their N-terminal lysine and other residues, thereby controlling chromatin structure and regulating cellular health and normal function [40]. These modifications mainly include acetylation, methylation, phosphorylation, ubiquitination, among others [35]. Each modification has a different effect on chromatin structure and gene expression. For instance, histone acetylation is usually associated with the loose structure of chromatin and promotes transcription factor binding and gene activation, whereas methylation may lead to gene activation or repression depending on the modification site [41, 42]. PTM maintains homeostasis in the body, which is maintained by histone-modifying enzymes such as histone acetyltransferases (HAT), histone deacetylases (HDACs), histone methyltransferases (HMT) and histone demethylases (HDM) [43]. When this balance is disrupted, uncontrolled gene expression can lead to diseases such as cancer. HAT can be mutated differently and is involved in all stages of development, from the growth and development of malignant tumors to their spread and invasion into target organs [44–46]. Studies have shown that mutations in the p300/CBP gene are associated with the development of various forms of leukemia and solid tumors [47–49]. In addition, GCN5-related N-acetyltransferases family (GNAT) members were found to be activated in colon, breast, and lung cancers, whereas p300/CBP-associated factor is usually reduced in ovarian, gastric, and esophageal cancers [50, 51]. In contrast, deacetylation, which is achieved by HDACs, causes chromatin to rearrange itself tightly, thereby suppressing gene expression. Therefore, excessive HDAC levels are strongly linked to the exacerbation of patients'disease and poor prognosis [52]. For example, overexpression or aberrant function of HDACs can be observed in colon, breast, and gastric cancers, which leads to the inactivation of tumor suppressor genes and thus promotes the proliferation and growth of cancer cells [53, 54]. In colorectal cancer cells, the expression of the cell-cycle protein-dependent kinase p21 is negatively correlated with the HDAC2 expression [55]. Methylation dysregulation has been shown to be associated with a negative correlation with HDAC2 levels [44]. Dysregulated methylation leads to hyperactivation of oncogenes or silencing of tumor suppressor genes.

As a class of RNA molecules that do not code for proteins, non-coding RNAs (ncRNAs) account for about 98% of the transcriptional output of the human genome, and play an important regulatory role in chromatin modification, gene transcription, cell proliferation, and differentiation [56–59]. Importantly, ncRNAs are often deregulated in cancer cells, resulting in either oncogenic or oncogenic roles, which are closely related to tumorigenesis and progression. Zhao *et al.* showed that miRNA-552 is an important oncogene that reduces the expression of PTEN, thereby aggravating the growth and metastasis of ovarian cancer [60]. Similarly, miR-155 is upregulated in a variety of cancers, such as breast, colorectal, and lung cancers, and promotes the proliferation and invasion of cancer cells [61]. On the contrary, let-7 is downregulated in a variety of cancers, and through the downregulation of oncogenes, such as KRAS that inhibit tumor progression [62].

**Fig. 1:**
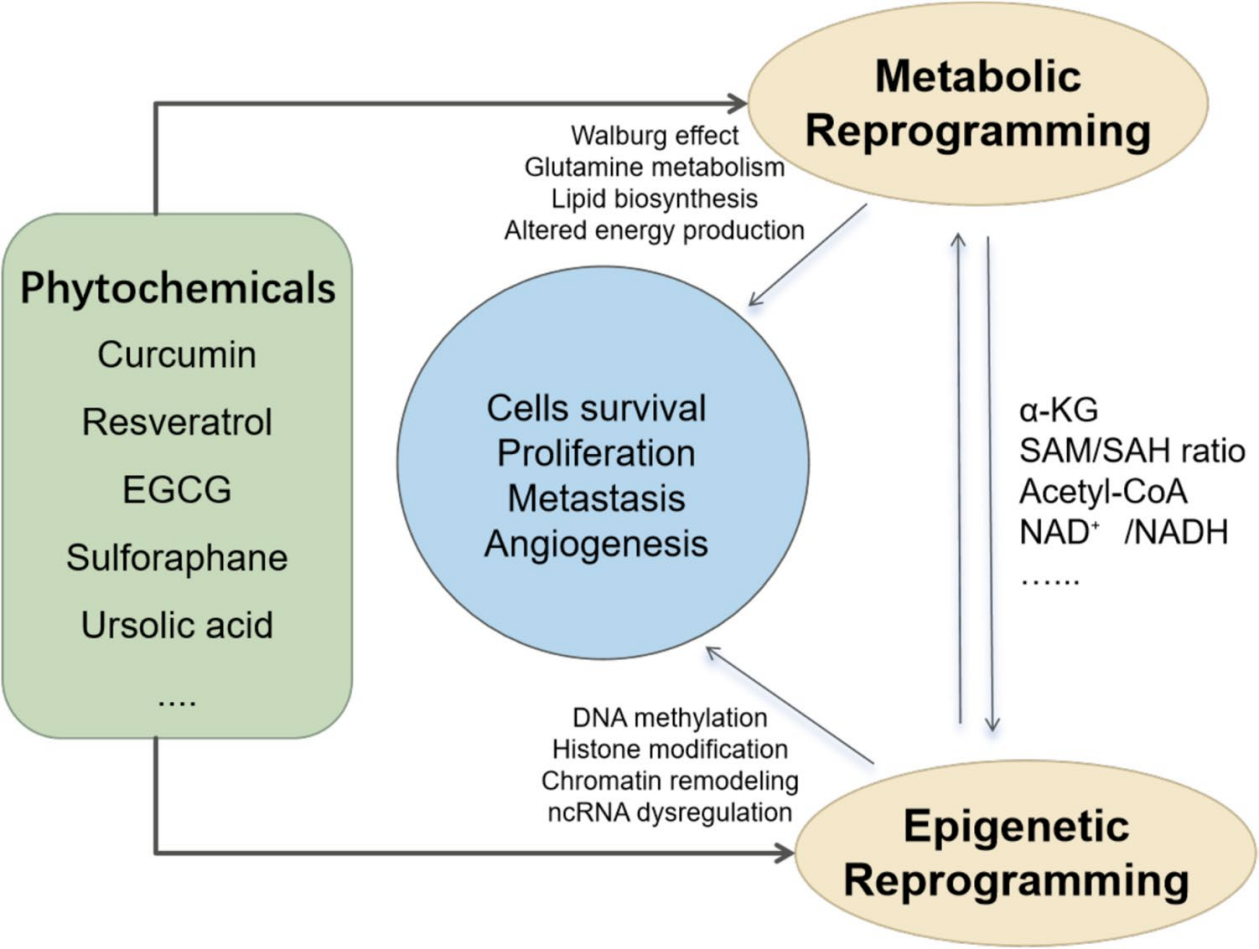
Phytochemicals Modulating Cancer Progression Through Metabolic and Epigenetic Reprogramming. EGCG, Epigallocatechin-3-gallate; α-KG, alpha-ketoglutarate; SAM/SAH ratio, S-adenosyl methionine/S-adenosylhomocysteine ratio; Acetyl-CoA, acetyl coenzyme A; NAD +/NADH, nicotinamide adenine dinucleotide oxidized (+)/reduced (H) form.

In the 1920 s, Otto Warburg discovered that cancer cells convert glucose to lactate through glycolysis even under aerobic conditions (the Warburg effect), which rapidly provides energy and creates an acidic environment that promotes tumor proliferation, invasion, and metastasis [63–65]. To meet the high-energy demand, cancer cells accelerate glycolysis through the upregulation of glucose transporter proteins (e.g., GLUT1) and the expression of key glycolytic enzymes (e.g., hexokinase) that accelerate glycolysis while relying on pentose phosphate pathway (PPP), the tricarboxylic acid (TCA) cycle, amino acid and lipid metabolism to support cancer cell growth [28, 66, 67]. Metabolic and epigenetic interactions form a complex network, such as TCA intermediates regulate DNA and histone methylation. In contrast, epigenetic alterations also feedback to influence metabolic pathways, e.g., changes in gene methylation enhance the expression of key enzymes of the PPP pathway and glycolysis, and histone modifications regulate lipid synthesis and one-carbon metabolism to meet the energy and biomolecular needs of cancer cells [68, 69]. This connectivity is critical for cancer progression and drug resistance, providing new insights and directions for combined metabolic-epigenetic anticancer therapies. Phytochemicals such as curcumin, resveratrol, EGCG, sulforaphane, and ursolic acid, among others, have been shown to simultaneously regulate metabolic and epigenetic pathways, thereby intervening in the energy metabolism and gene expression patterns of cancer cells. These natural compounds act as regulatory bridges between metabolic reprogramming and epigenetic regulation by targeting key metabolic enzymes and epigenetic regulatory factors, thereby influencing cancer cell proliferation, migration, and drug resistance (Fig. 1). By affecting these key pathways, phytochemicals hold promise as potential strategies for cancer therapy. Here, we will summarize the changes in epigenetic and metabolic pathways and the multiple effects of phytochemicals in different cancer progression models (Table I).

**Table I:** The Alteration in Various Cancers and the Anticancer Effects of the Dietary Phytochemicals Through Regulation of Epigenetics and Metabolism

Cancer Type	Mechanism Category	Signaling Pathway/Alteration	Phytochemicals and Their Anti-Cancer Effects	Reference
**Colorectal Cancer**	Signal Pathway Regulation	Upregulation of Wnt pathway, EGFR, P53, TGF-β mutations; KRAS induces GLUT1 and ASNS expression	1. **Curcumin**: Downregulates pathway-related gene expression (e.g., DLEC1), inhibits Wnt pathway	[70]
			2. **SFN**: Inhibits HDAC3, activates NRF2 signaling, reduces GLUT1 expression, inhibits glycolysis	[71]
	Epigenetic Regulation	APC gene mutation; BRAF, TIGAR abnormalities	1. **Curcumin**: Induces methylation exchange, reduces DNA methyltransferase binding and histone acetylation/methylation	[70]
			2. **Resveratrol**: Upregulates anti-cancer miRNA (miR-101b), downregulates IL-6 and TNF-IL	[72]
	Metabolic Reprogramming	KRAS mutation reprograms carbohydrate, amino acid, and lipid metabolism	1. **Soyasaponin I (SsI)**: Regulates amino acid metabolism and estrogen signaling pathways and binds to DNMT1 to inhibit proliferation	[73]
**Lung Cancer**	Signal Pathway Regulation	PI3K/Akt/mTOR, HIF-1α abnormalities	1. **Curcumin**: Inhibits HIF-1α signaling, reduces glycolysis and ROS production	[74]
			2. **Resveratrol**: Regulates Ca^2^⁺/CaMKKB/AMPK pathways, inhibits glucose uptake and glycolytic enzymes GLUT1 and PFK1	[75]
	Epigenetic Regulation	CpG island hypermethylation, histone H4 modifications	1. **Curcumin**: Inhibits DNMT and HDAC, regulates histone modifications	[76]
			2. **EGCG**: Downregulates DNMT1, reduces DNA methylation, reactivates tumor suppressor genes (e.g., p16)	[77]
	Metabolic Reprogramming	Abnormal palmitic acid and bile acid metabolism	1. **Quercetin**: regulates the expression of tobacco carcinogen metabolism genes and inhibits lipid metabolism pathways	[78]
**Skin Cancer**	Signal Pathway Regulation	Abnormalities in NRF2 and KEAP1 pathways	1. **SFN**: inhibits tumor metastasis through the NRF2 pathway and reduces H3K27me3 levels	[79]
	Epigenetic Regulation	RASSF1A methylation; H3K9 and H3K27me3 abnormalities	1. **EGCG**: reduces DNA methylation levels in skin cancer cells and increases histone acetylation	[80, 81]
			2. **Silymarin**: reduces UVB-induced DNA damage and delays tumorigenesis	[82]
**Prostate Cancer**	Signal Pathway Regulation	PI3K/Akt/mTOR, Wnt/β-catenin	1. **Curcumin**: Inhibits PI3K/Akt/mTOR and Wnt/β-catenin signaling pathways, regulates metabolic gene expression	[83]
	Epigenetic Regulation	GSTP1 promoter hypermethylation; EZH2-mediated H3K27me3 abnormalities	1. **SFN**: Induces histone acetylation to activate promoter demethylation and regulate the cell cycle	[84]
			2. **Curcumin**: Reverses Nrf2 hypermethylation, reduces m6A modification enzymes (e.g., METTL3)	[85]
	Metabolic Reprogramming	Androgen metabolism disorder; increased fatty acid synthesis	1. **UA**: Regulates lipid metabolism-related genes, reduces tumor cell growth	[86]
			2. **DATS**: Induces ROS signaling to suppress tumor growth	[87]
**Breast Cancer**	Signal Pathway Regulation	GPR30-Akt signaling; BRCA1 inhibition	1. **Genistein**: Inhibits GPR30-Akt pathway, regulates cell cycle-related ROS accumulation	[88]
	Epigenetic Regulation	Overexpression of miR-25; BRCA1 methylation	1. **EGCG**: Downregulates miR-25, inhibits tumor growth	[89]
			2. **Soyflavone**: Inhibits ERα/c-erbB-2 expression, activates tumor suppressor PTEN	[90]
	Metabolic Reprogramming	Abnormal glycolysis; prolactin-related metabolism	1. **EGCG**: Inhibits tumor cell growth via glucose metabolism pathways	[91]
			2. **Combination Therapy (Curcumin + Genistein)**: Synergistically inhibits tumor cell proliferation and promotes apoptosis	[92]

## Colorectal Cancer

According to the U.S. Cancer Statistics Working Group of Centers for Disease Control and Prevention and National Cancer Institute, the latest report in 2021 delineated 36 new colorectal cancer (CRC) cases were reported per 100,000 people, ranking the fourth place in the top 10 cancers of new cancer cases over all races, ethnicities, and genders nationwide.

The progression of CRC is characterized by a sporadic and stepwise development, known as the adenoma-carcinoma sequence. In this cancer-driven sequence, the accumulation of mutations in multiple signaling pathways is consistently observed, including Wnt, EGFR, P53, and TGF-β. Notably, early mutations in the adenomatous polyposis coli (APC) gene and the BRAF oncogene initiate the development of serrated polyps which are responsible for most CRC. Preclinical studies support that upregulation of the Wnt pathway following APC deletion induces expression of TP53-inducible glycolysis and apoptosis regulator (TIGAR) and RAC1. TIGAR regulates the regeneration of glutathione (GSH) levels. RAC1, conversely, is involved in ROS production for cell signaling and proliferation. Activating the Wnt pathway regulates cellular oxidative stress to aid cell proliferation [93]. The evidence shows that KARS mutation contributes to the metabolic reprogramming in CRC to support cancer cell growth by rewiring carbohydrate, amino acid, and lipid metabolisms. GLUT1 is upregulated to facilitate glucose uptake by KRAS mutation [94]. Additionally, KRAS mutation induces the upregulation of asparagine synthetase (ASNS) to enhance de novo asparagine biosynthesis and regulate cellular adaption to glutamine depletion [95].

Phytochemicals perform the anti-oxidant and anti-inflammatory capacities via epigenetic modulation. For example, phytochemicals are reported to revoke the hypermethylated state of the NRF2 promoter region, a key regulator in the NRF2-ARE signaling pathway, in cancer cells and to hyper-methylate inflammation-associated genes to downregulate the expression [96]. In human colon cancer HT29 cells, curcumin (CUR) demethylated the promoter region of DLEC1 and reduced DNA methyltransferases and histone deacetylases at the protein level [97]. Through analyzing methyl-seq and RNA-seq data of AOM/DSS mouse model, Guo *et al*. observed that curcumin hypomethylated inflammation-associated genes, including Duoxa2, Gja1, Icam1, Igfbp4, Itgb2, Lgals9, and Pf4, which were upregulated by AOM/DSS [98]. The anti-inflammatory effects of resveratrol through epigenetic regulation were reported in Altamemi’s study [99]. In the DSS-induced colitis mouse model, resveratrol treatment elevated the anti-inflammatory miRNAs, miRNA-101b and miRNA-455, while downregulating IL-6 and TNF-α [99]. SFN, a well-characterized HDAC inhibitor and Nrf2 activator, has been demonstrated to reduce the expression of HDAC3, P300/CBP-related factors, KAT2A/GCN5, and DNMT1 in HCT 116 and SW8409 cells following SFN treatment [100]. Notably, SFN treatment also demethylates the Nrf2 promoter region in Caco-2 cells, thereby activating the NRF2 signaling pathway. Furthermore, SFN exhibits inhibitory effects on DNMT activity [101]. Soyasaponin I (SsI), an oleanane triterpenoids and a prominent component of Group B of soyasaponins, has been demonstrated to exhibit biological activities associated with pulses, including anti-inflammatory effects and the inhibition of the migration of cancer cells [102–107]. Xia *et al*. reported that SsI exhibited inhibitory effects on the proliferation of HCT116 and LoVo cells, with IC50 values of 161.4 µM and 180.5 µM, respectively. Furthermore, metabolomic analysis revealed that SsI significantly impacted the absorption and metabolism of amino acids in HCT116 cells. Additionally, molecular docking analysis elucidated the mechanism of SsI-mediated inhibition of HCT116 proliferation, suggesting that this inhibition may be attributed to the binding of SsI to DNMT1 and ERK1. The findings of this study indicate that SsI inhibits the growth of HCT116 cells by regulating amino acid metabolism and the estrogen signaling pathway [108].

Multiple signaling pathways and transcription factors are involved in regulating glycolysis in CRC, including PI3K/AKT/mTOR, AMPK, HIF-1, and c-myc. Several phytochemicals have been studied on the anti-CRC effect via either directly or indirectly regulation of glycolysis [109]. Anthocyanin and silybinare flavonoids, which downregulate the expression of GLUT1 in MC38 cells and LoVo cells, respectively, to reduce glucose uptake and affect glycolysis [110, 111]. Kaempferol inhibits CRC glycolysis in HCT116 cells and DLD1 cells by increasing expression of miR-339-5p and decreasing levels of hnRNPA1 and PTBP1. As a result, PKM2 expression is downregulated [112]. Wogonin inhibits glucose uptake and lactate production in HCT116 cells under hypoxia by downregulating HIF-1αexpression and transcriptional activity of PI3K, and AKT, leading to a decrease in glycolytic enzymes, including HK2, PDHK1, and LDHA [113]. Resveratrol would enhance pyruvate dehydrogenase (PDH) complex activity, elevates oxidative capacity, and suppresses glycolysis in Caco2 and HCT116 cells by probing and modulating the Ca^2+^/CaMKKB/AMPK pathway [114].

## Prostate Cancer

Prostate cancer (PCa) accounts for 29% of all cancer diagnoses and 20% of cancer-related deaths in men in the United States in 2024 [115]. Progression from prostate intraepithelial neoplasia to androgen-independent invasive carcinoma is driven by multiple epigenetic and metabolic alterations [12].

Hypermethylation of the promoter region of the GSTP1 gene occurs in approximately 90% of primary prostate cancers, leading to silencing of this tumor suppressor gene. This is an early and common epigenetic change that serves as a diagnostic marker [116]. In addition, EZH2-mediated trimethylation of H3K27 is significantly increased in advanced metastatic prostate cancers, which promotes cell proliferation and suppresses tumor suppressor gene expression [117]. Non-coding RNAs such as miR-101 deletion leads to EZH2 overexpression, which further accelerates cancer progression, whereas dysregulation of the chromatin remodeling factor SWI/SNF complex is closely associated with the emergence of desmoplasia-resistant prostate cancer and a neuroendocrine phenotype [118, 119]. These alterations not only reveal the molecular mechanisms of prostate carcinogenesis, but also provide potential targets for diagnosis and treatment [116].

In TRAMP-C1 cells and TRAMP mouse models, curcumin and its analog FN1 reversed the hypermethylation status of the Nrf2 gene and significantly activated the anti-oxidative stress pathway [120]. Furthermore, in PC3 and DU145 cells, curcumin sensitized cancer cells to radiation therapy by activating miR-143 expression while inhibiting radiation-induced autophagy [121]. Curcumin also modulated the JNK pathway by inhibiting the level of the epigenetic marker H3K4me3, and showed significant anti-tumor effects in LNCaP cells and xenograft mouse models when combined with the epigenetic inhibitor JQ-1 [122]. In addition, curcumin reduced tumor cell proliferation and migration by inhibiting m6A modification-related enzymes (e.g., METTL3) and regulating the expression of cyclic RNA circ0030568 in PC3 cells [123]. Curcumin reduced the nuclear transcriptional activity of β-catenin and the expression of energy metabolism-related genes in LNCaP and C4-2B cells by inhibiting the PI3K/Akt/mTOR and Wnt/β-catenin signaling pathways [83]. Further studies have shown that curcumin significantly increased ROS production in PC3 cells, while interfering with glutamine metabolism and glucose metabolism, thereby suppressing metabolic homeostasis in tumor cells [124]. In TRAMP mice, curcumin interfered with androgen metabolism by inhibiting the expression of AKR1C2 and delayed tumor progression [125]. Curcumin also further enhanced its anti-tumor effect by down-regulating metabolism-related proteins and enzymes (e.g., cell cycle protein D1 and GSK3β) in combination with epigenetic regulatory mechanisms [126].

On the other hand, SFN activates antioxidant genes (e.g., NQO-1) and inhibits the proliferation and survival of prostate cancer cells by decreasing the expression of DNMT and HDAC and by promoting demethylation of key gene promoters and histone acetylation [84]. It has been shown that in prostate cancer cell lines, SFN inhibits the expression and activity of telomerase reverse transcriptase (hTERT) and regulates epigenetic processes by enhancing the acetylation of histone H3 lysine 18 and dimethylation of H3K4 [127]. Notably, SFN also induced S-phase and G2/M-phase cell cycle arrest in DU145 and PC3 cells, which was accompanied by a significant increase in the acetylation of histones H3 and H4, affecting cell cycle regulatory proteins and signaling pathways [128]. It was reported that SFN could modulate the pattern of DNA methylation and significantly reduce metabolic markers (e.g., fatty acids, acetylated fatty acids, and acetylated fatty acids) in plasma and prostate tissues, thereby inhibiting the development of prostate cancer in the TRAMP mouse model [129, 130].

Meanwhile, ursolic acid (UA) reversed the differentially methylated region (DMR) triggered by PTEN loss in a PTEN-deficient mouse model and regulated the methylation of CpG sites associated with inflammatory pathways (e.g., NF-κB and IL-6) [131]. This suppresses the expression of pro-oncogenes (e.g., Has3, Cfh, and Msx1) while upregulating tumor suppressors (e.g., BDH2). At the metabolic level, UA intervenes in lipid metabolism by inhibiting fatty acid synthase (FASN) and acetyl-CoA carboxylase (ACC), thereby reducing the supply of lipids required for cancer cell proliferation [132]. Garlic-derived trisulfide DATS induces apoptosis and downregulates the expression of the tumor marker Ki-67, through inhibition of tumor growth and reduction of tumor volume and weight [87]. Its anticancer mechanisms include activation of the P38/JNK/MAPK pathway, downregulating Nrf2/Akt signaling, decreasing the expression of X-linked inhibitor of apoptosis protein (XIAP), and inhibiting p-STAT3, which reduces malignant proliferation and tumorigenicity of tumors [133, 134]. In addition, DATS induces iron death targeting cancer cells by overcoming apoptosis resistance [135].

## Lung Cancer

Lung cancer, a leading public health problem is resultant from the interactions of genetic, epigenetic, metabolic and environmental factors. Promoter-specific hypermethylation of the unmethylated CpG island of several genes such as CDKN2A, methylguanine-methyltransferase (MGMT), RARb, APC and RASSF1 is the strikingly less invasive epigenetic biomarker which can be detected in early lung tumors, especially in bronchoscopic washings/brushings, sputum aspirates, and blood [136–139]. In addition, aberrant histone H4 modification patterns, such as hyperacetylation of H4K5/H4K8, hypoacetylation of H4K12/H4K16, and lack of trimethylation of H4K20 are important biomarkers to diagnose early stages of lung cancer [140]. Metabolomics has been shown to be a powerful tool for determining early-stage biomarkers of lung cancer based on plasma metabolite profiling [141]. Over 150 metabolites are found to be associated with lung cancer [142]. A non-invasive metabolomic analysis of biofluids has revealed that purines/pyrimidines and proteins have enhanced dysregulation than other classes of metabolites in early stage non-small cell lung cancer (NSCLC) post-surgical resection [143]. Aberrant bile acid metabolism has been associated with migration of lung adenocarcinoma [144]. Metabolites like palmitic acid, heptadecanoic acid, 4-oxoproline, tridecanoic acid, and ornithine have been considered for early lung cancer screening [145]. Sphingomyelin (d18:0/22:0), a sphingolipid, and taurodeoxycholic acid 3-sulfate are also identified as early biomarkers of lung cancer [146]. It is interesting to note that certain metabolites activate a series of signal transduction pathways and affect epigenetics via metabolite sensing mechanisms [147].

Medicinal plant extracts and their constituents have shown promising therapeutic value against lung cancer. Anti-cancer effect of curcumin is well known. This dietary chemopreventive phytochemical is known to inhibit angiogenesis and metastasis by epigenetic modifications via inhibition of DNMTs and HDACs. It also interferes with cancer cell growth and proliferation by altering mitochondrial energy metabolism thereby reducing oxidative stress via inhibition of FoF1-ATP synthase [76]. The beneficial effects of ginseng are studied in various health conditions. It is shown to have the potential to synergize immunotherapy in lung cancer [148]. Ginseng and its components including total ginsenoside and ginseng polysaccharide are found to regulate important targets related to lung cancer proliferation, migration, and apoptosis, such as JUN, IL-1β, IL-2, ICAM1, HMOX1, MMP9, MMP2, PTGS2, and TNF [148]. Interestingly, ginseng’s anti-cancer effect has been correlated with epigenetic methylation of immune response-related genes and upregulation of gut microbial metabolite valeric acid and downregulation of L-kynurenine [148, 149]. A metabolic study of EGCG in A549 cells has identified 33 differentially expressed metabolites [150]. In an NNK-induced in-vivo model of lung cancer, EGCG regulated epigenetic mechanism by downregulating DNMT1 along with phospho-histone H2AX (γ-H2AX) and p-AKT reduction [77]. Resveratrol has shown promise in reducing tumor weight, volume, and metastasis while inducing apoptosis in lung carcinoma *in vivo* model [151]. Its therapeutic capacity is shown to decrease glucose uptake and glycolysis with potential targets being Glut1, PFK1, HIF-1α, ROS, PDH, and the CaMKKB/AMPK pathway [152]. Quercetin is another chemopreventive agent that is found to suppress metastatic behavior of lung cancer via suppression of Snail‐mediated EMT [153]. Metabolomic studies have shown that dietary quercetin modulated the expression of genes involved in the metabolism of tobacco carcinogens in humans [78].

## Skin Cancer

The skin is the organ that is the most exposed to the external environment, and its integrity and the extent of interaction with environmental factors affect the health of the organisms including human. The global incidence of skin cancers such as melanoma, basal cell carcinoma and squamous cell carcinoma is continuously increasing [154]. Ultraviolet-B (UVB) radiation, air pollutants like black carbon and PM10 are the primary factors for the initiation of cutaneous malignancy [155, 156]. Abnormal DNA methylation is crucial in regulating melanoma gene expression. Hypermethylation of the RASSF1A gene is strongly associated with melanoma progression, whereas hypomethylation of the Alu and LINE-1 sequences is associated with transcriptional silencing of cancer-related genes [157]. In addition, aberrant histone modifications significantly affected gene expression, and the synergistic effect with DNA methylation further emphasized the potential significance [158, 159] Histone methyltransferase SETDB1 promotes melanoma progression and suppresses target gene expression through trimethylation of H3K9, revealing an important interaction between epigenetic modifications and gene mutations in melanoma development [160].

Isothiocyanates (ITCs), notably SFN, exhibit potent chemopreventive effects in UV- and chemical-induced skin carcinogenesis. SFN inhibits PcG proteins like Bmi1 and EZH2, reducing H3K27me3 levels, and thus blocking tumor progression [79]. In squamous cell carcinoma, SFN reduces arginine methylation at H3, suppressing tumor growth and metastasis [36]. Our previous study demonstrated that SFN promotes Nrf2 signaling and attenuates oncogenic changes through various mechanisms, such as CpG demethylation and down-regulation of DNMT and HDAC, and inhibition of KEAP1 degradation [161, 162]. EGCG has been shown to regulate epigenetic processes in skin cancer by reducing DNA methylation in SCC-13 and A431 skin cancer cell lines through DNMT suppression [80]. Additionally, applying EGCG topically has been observed to counteract UVB-induced global hypomethylation in the SKH-1 hairless mouse model [81]. EGCG has been found to reactivate tumor suppressor genes like cip1/p21 and p16INK4a in human skin cancer cells by reducing DNA methylation and increasing histone acetylation [80]. Moreover, EGCG can demethylate the RECK promoter, restore its transcription, and increase the levels of tumor suppressors (e.g., p21, p16, and p53) in melanoma cells by inhibiting class I HDAC, thereby exerting an antitumor effect [163].

In DMBA-TPA and DMBA-MEZ SENCAR mouse models, silymarin significantly delays tumor onset, reduces tumor incidence, multiplicity, and volume, and promotes tumor regression through cell proliferation inhibition and apoptosis induction in tumor cells [82]. Silibinin demonstrated potent inhibition of UVB-induced thymine dimer formation in SKH-1 hairless mouse skin epidermis, as evidenced by immunostaining. Additionally, dietary silibinin also reduced UVB-induced thymine dimer-positive cells in mouse skin epidermis. These results suggest that silibinin's molecular interaction not only prevents UVB-induced DNA damage in mouse skin, but also potentially contributes to its inhibitory effect on UVB-induced skin tumor initiation [164].

Our previous studies have shown that UA reduces tumor volume and number by activating antioxidant, anti-inflammatory, and anticancer pathways through targeting Nrf2, cell cycle protein D1, and inflammation-related genes [165, 166]. Delphinidin significantly inhibited TPA-induced tumor transformation (69.4% to 99.4%) by promoting the expression of the Nrf2 pathway and its downstream target genes (e.g., HO-1), inducing promoter demethylation, and inhibiting the expression of DNMT and HDAC [167].

## Breast Cancer

According to previous studies, the carcinogens used for breast cancer models are typically 7,12-dimethylbenz[a]anthracene (DMBA), 17β-estradiol (E2), and N-nitroso-N-methylurea (NMU) [168–170]. In recent years, due to the resistance of advanced metastatic breast cancer to radiation and chemotherapy, many researchers have shifted their focus to the early prevention of breast cancer. Studies have identified several key preventive compounds, including (1) EGCG, (2) genistein, (3) soy flavonoids, and (4) resveratrol. The inhibitory effect of EGCG on tumor glucose metabolism was elucidated in a study by Wei *et al* [91]. EGCG activated all three cystatinases in a concentration-dependent manner and up-regulated the expression levels of a variety of pro-apoptotic genes such as puma, caspase3, caspase8, among others. In addition, EGCG decreased the expression of the antiapoptotic genes survivin, bcl-2, bcl-xl, and c-myc [91].

Previous studies have demonstrated that miR-25 is an overexpressed gene in breast cancer tissues and that miR-25 levels are elevated in human serum [171]. The results of a series of experiments showed that EGCG was able to down-regulate the expression level of miR-25, and knockdown of miR-25 further revealed a significant inhibitory effect on the proliferation of MCF-7 breast cancer cells and promoted the occurrence of apoptosis [89].

Genistein, the major isoflavone in soy, prevents breast cancer by inhibiting the GPR30-Akt signaling pathway [88]. It has been found that in triple-negative breast cancer (TNBC) cells, genistein regulates the cell cycle by down-regulating BRCA1 and activating Nrf2, which induces the accumulation of ROS [172]. In addition, genistein prevents breast cancer by inhibiting DNMT1, reactivating the expression of tumor suppressor genes (TSGs), such as PTEN, APC, and stratifin [173–176]. In rat model experiments, genistein flavonoid significantly inhibited N-methyl-N-nitrosourea (MNU)-induced carcinogenesis in a dose-dependent manner [168].

Soy flavone (7,4′-dihydroxyisoflavone), has also shown breast cancer chemopreventive effects [177]. It inhibited the accumulation of ceruloplasmin in the presence of estradiol and potentiated the anticarcinogenic effect of paclitaxel [178]. It was demonstrated that soy flavone significantly up-regulated the expression of estrogen-responsive gene, pS2, and simultaneously increased the level of ROS in tumor cells and downregulated the expression of ERα and c-erbB-2 in a dose-dependent manner [90]. Due to the structural similarity between genistein and soy flavonoids, the study further tested the combined inhibitory effect of both compounds. In female Big Blue transgenic rats, the combined application of genistein and soy flavones in DMBA-driven model significantly increased cell proliferation, apoptosis, and was more potent compared to genistein alone. This suggests a potential synergistic anticancer effect of genistein and soy flavones [92].

## Conclusion and Future Perspectives

Factors such as genetic mutations, dysregulation of cellular metabolism, epigenetic alterations, and environmental factors not only influence the development of cancer, but also provide potential targets for cancer therapy. In recent years, phytochemicals have received widespread attention for their anticancer potential. Phytochemicals, including flavonoids, phenols and alkaloids, are widely found in people's daily diet. Many studies have shown that these substances are able to prevent the development of cancer and inhibit the proliferation and metastasis of cancer cells through a variety of mechanisms. For example, curcumin reverses the hypermethylation status of the Nrf2 gene; resveratrol promotes apoptosis by regulating the p53 signaling pathway; and sulforaphane restores the expression of antioxidant genes by inhibiting histone deacetylase and DNA methyltransferase. The bioavailability of phytochemicals largely determines their potency and anticancer effects. Many phytochemicals exhibit low bioavailability, and absorption and distribution are influenced by a variety of physicochemical properties as well as physiological factors. Curcumin, tea polyphenols, and anthocyanins have shown strong potential in anticancer studies but have poor solubility and bioavailability. These substances are rapidly metabolized and excreted in the intestine or urine, making it difficult to reach effective plasma concentrations and limiting their anticancer effects. Many studies have attempted to improve the bioavailability of phytochemicals through drug delivery systems, such as nanocarriers and liposomes. In addition, the action of phytochemicals in the body may be affected by a wide range of factors, such as individual differences, lifestyle, and genetic background, leading to differences in their action in different populations. Although the anticancer potential of phytochemicals is widely supported in experimental and epidemiologic studies, significant challenges remain in their transition from basic research to clinical applications. Overall, phytochemicals have great potential for cancer prevention and treatment, and future research should focus on improving the bioavailability of phytochemicals, exploring their synergistic mechanisms of action in depth, validating their clinical efficacy, and developing therapeutic strategies for anticancer prevention and treatment that target epigenetics and metabolism. By addressing these challenges, phytochemicals are expected to become effective tools for cancer preventive therapy.

## Abbreviations

5-mC: 5′-CpG-3′ dinucleotide endocytosine
γ-H2AX: Phospho-histone H2AX
ACC: Acetyl-CoA carboxylase
APC: Adenomatous polyposis coli
ASNS: Asparagine synthetase
CRC: Colorectal Cancer
CUR: Curcumin
DMBA: 7,12-Dimethylbenz[a]anthracene
DMR: Differentially methylated region
E2: 17β-estradiol
EGCG: Epigallocatechin gallate
FASN: Fatty acid synthase
GFs: Genistein flavonoids
GSH: Glutathione
HAT: Histone acetyltransferases
HDACs: Histone deacetylases
HDM: Histone demethylases
HMT: Histone methyltransferases
hTERT: Telomerase reverse transcriptase
ITCs: Isothiocyanates
MGMT: Methylguanine-methyltransferase
MNU: N-methyl-N-nitrosourea
ncRNAs: Non-coding RNAs
NMU: N-nitroso-N-methylurea
NSCLC: Non-small cell lung cancer
PCa: Prostate cancer
PDH: Pyruvate dehydrogenase
PPP: Pentose phosphate pathway
PTMs: Post-translationally modifying histones
SFN: Sulforaphane
SsI: Soyasaponin I
TCA: Tricarboxylic acid
TIGAR: TP53-induced glycolysis and apoptosis regulator
TNBC: Triple-negative breast cancer
TSGs: Tumor suppressor genes
UA: Ursolic acid
UVB: Ultraviolet-B
XIAP: X-linked inhibitor of apoptosis protein
